# Quality of Life of Patients With Multi-Organ Autoimmune Disease and Its Relationship to Patient-Centered Care: Protocol for a Longitudinal Cohort Study

**DOI:** 10.2196/77786

**Published:** 2026-03-09

**Authors:** Marianne Bayrhuber, Christian Schlett, Julia Slesaczeck, Sabrina Herbst, Gabriele Mueller, Alexandra Nieters, Bodo Grimbacher, Erik Farin-Glattacker

**Affiliations:** 1 Faculty of Medicine and Medical Center, Section of Health Care Research and Rehabilitation Research, Institute of Medical Biometry and Statistics, Albert-Ludwigs-University of Freiburg, Hugstetter Str. 49, Freiburg, 79106, Germany, 49 761 27083732; 2 Center for Evidence-Based Healthcare, Faculty of Medicine and University Hospital Carl Gustav Carus, TUD Dresden University of Technology, Dresden, Germany; 3 Institute for Immunodeficiency, Center for Chronic Immunodeficiency (CCI), Medical Center, Faculty of Medicine, Albert-Ludwigs-University of Freiburg, Freiburg, Germany; 4 Clinic of Rheumatology and Clinical Immunology, Center for Chronic Immunodeficiency (CCI), Medical Center, Faculty of Medicine, Albert-Ludwigs-University of Freiburg, Freiburg, Germany; 5 DZIF – German Center for Infection Research, Satellite Center Freiburg, Freiburg, Germany; 6 RESIST – Cluster of Excellence 2155 to Hanover Medical School, Satellite Center Freiburg, Freiburg, Germany; 7 CIBSS – Centre for Integrative Biological Signalling Studies, Albert-Ludwigs University Freiburg, Freiburg, Germany

**Keywords:** health-related quality of life, multi-organ autoimmunity, patient preferences, rare disease, health status

## Abstract

**Background:**

Multi-organ autoimmunity belongs to a group of ultrarare diseases characterized by complex autoimmune or autoinflammatory processes affecting multiple organs. In addition to adequate medical care for patients with multi-organ autoimmunity, the understanding of patients’ treatment preferences and the measurement of their health-related quality of life are essential for improving therapy for these patients.

**Objective:**

This study has three goals: (1) to compile a modular questionnaire package using established instruments to assess health-related quality of life and health status; (2) to develop, through a participatory process, and conduct psychometric testing of a new self-report questionnaire assessing patients’ treatment preferences; and (3) to evaluate quality of life and patients’ preferences in a longitudinal study.

**Methods:**

To address our goals, we are conducting a mixed methods study involving 300 adult patients undergoing treatment for multi-organ autoimmune diseases; the study includes qualitative interviews with patients and their physicians and a quantitative longitudinal study with 4 measurement time points (at study enrollment and at 3, 6, and 9 months later). We will address our first goal by conducting psychometric analyses to assess the applicability of the modularized questionnaire package to our target population. We will determine item characteristics and conduct exploratory factor analyses and internal consistency analyses with the scales used to assess generic and disease-specific quality of life, as well as disease-specific health status. To address our second goal, we will conduct (1) qualitative interviews with patients and their physicians to explore the preferences of our target population, (2) a 2-round Delphi study to select preferences that are highly relevant to most patients, (3) cognitive pretests to ensure the acceptability and comprehensibility of the scale items and instructions, and (4) psychometric analyses to guide item selection and evaluate the reliability and validity of the final scales.

**Results:**

This paper describes the protocol for the longitudinal cohort study of the research project Qualy-GAIN (German Multi-Organ Auto-Immunity Network), which was funded from January 2023 to December 2025. Data were collected at 4 measurement points from July 2024 to June 2025. First results are expected to be submitted for publication in spring 2026.

**Conclusions:**

A result of this study will be a package of questionnaires that can be used to assess health-related quality of life, disease-specific health status, and treatment preferences. Due to the voluntary nature of participation and the low burden associated with participation, we believe that the benefits of the study outweigh potential risks. In case the questionnaires evaluated in this study prove to be reliable, valid, and useful in practice, they can be transferred to the routine treatment of patients with multi-organ autoimmune diseases to improve treatment and promote patient-centered care.

## Introduction

Patients with multi-organ autoimmunity belong to a group of patients with ultrarare diseases and are characterized by complex autoimmune or autoinflammatory processes affecting several organs. Multi-organ autoimmunity is often caused by rare genetic mutations that are categorized as congenital defects of immunity and can lead to various chronic disease manifestations [1]. However, multi-organ autoimmune diseases do not typically result in immediate mortality. Instead, affected individuals experience prolonged disease burden, extensive use of health care resources, and frequent consultations with multiple specialists (“physician hopping”) while seeking optimal treatment.

Patient-reported outcome measures (PROMs) in health care can inform the treatment team about the patients’ quality of life (QoL) at an individual level and can be used to monitor the treatment process and improve communication and shared decision-making [2]. At the system level, this information enables statements about the effectiveness and feasibility of care services, thus creating the prerequisite for improved adaptation processes to patients’ needs. The QoL of patients with chronic diseases is not only influenced by pathophysiology and physical symptoms but also partly by the organization of treatment and the physician-patient relationship and interaction [3,4]. The dynamics of physician-patient communication, especially the decision-making process related to therapeutic choices, greatly influence patient adherence and satisfaction [5,6]. Patients with inflammatory rheumatic diseases in general prefer to have more information from their health care providers. However, lower-educated, older patients with low health literacy and high confidence in their health care providers prefer to leave treatment decisions to their physicians [3]. In addition to adequate medical care for patients with multi-organ autoimmunity, understanding patient preferences during interactions with physicians and the measurement of patients’ QoL are essential for improving therapy [2,3]. Medical care for patients with autoimmune diseases can be improved when patient preferences and QoL are taken into account. By considering individual preferences, such as treatment goals and communication styles, health care providers can create more personalized and effective treatment plans [2]. This patient-centered approach can improve adherence to treatment and enhance overall patient satisfaction and long-term outcomes. However, most research focuses on clinical outcomes and health-related QoL, whereas patient preferences and satisfaction and their fulfillment are less studied [7].

The collection of patient-reported experience measures, that is, the fulfillment of patient preferences, also makes it possible to examine process indicators of medical care. Currently, no published research has examined the QoL of patients with multi-organ autoimmunity and its relationship to patient-centered care. In this understudied but important area, we will pursue 3 goals.

First, we will compile a modular questionnaire package of established instruments to assess QoL and disease-specific health status (eg, perceived symptoms, fatigue, and disease-related restrictions in activity and participation). The questionnaire package must meet the following requirements: (1) it should reflect the heterogeneity of different autoimmune diseases and their consequences for patients’ health status in a meaningful way (a modular structure is planned for this purpose), (2) it should have a high methodological quality in our target group (reliability and validity), (3) it should be accepted by the patients, and (4) it should be informative for treatment practice.

Second, we will undertake the participatory development and psychometric testing of a new self-report questionnaire to assess (1) patients’ preferences concerning patient-physician communication and interaction (eg, shared decision-making; microlevel), (2) preferences regarding the mode of cooperation between physicians (including diagnostic procedures and cocare; mesolevel), (3) preferences regarding the support system (including patient self-help; macrolevel), and (4) perceived fulfillment of these preferences. This instrument, which is to be newly developed according to psychometric criteria, is intended to capture a central aspect of patient-centered treatment, namely, the consideration of patient preferences in everyday care.

Third, we will use newly developed questionnaires in a patient sample of approximately 300 individuals in a prospective, longitudinal, epidemiological study with 4 measurement time points. In this study, we will test the following three hypotheses:

1. Fulfillment of preferences at an earlier time point is associated with better self-reported health status and improved health-related QoL at a subsequent time point.
2. There are plausible correlations between patients’ self-reported health status and QoL, and pathophysiology and physical symptoms. Subjective health status can only partly be predicted by objective parameters, resulting in the need to consider it as an independent end point of treatment.
3. The feedback of the collected patient data on self-reported health status, QoL, and fulfillment of preferences to their physicians is considered useful by the treatment team to optimize treatment and, consequently, leads to more patient-centered care.

The identification of patient preferences and their relationship with patients’ subjective health status and QoL offers a new perspective on personalized care approaches for patients with multi-organ autoimmune diseases. The newly developed questionnaire can be used in routine care for patients with multi-organ autoimmune diseases to optimize their treatment. Furthermore, the procedure proposed here and the modular questionnaire set may be transferred to studies involving patients with other rare diseases.

## Methods

### Study Design

To address our goals, we will first conduct preliminary studies that include qualitative interviews with patients and physicians to explore patient treatment preferences, a 2-round Delphi study to select items that are highly relevant to most patients, and cognitive pretests to ensure the acceptability and comprehensibility of the questionnaire. Second, we will conduct an epidemiologic health services research cohort study with prospective longitudinal data collection at 4 measurement time points (at study enrollment and at 3, 6, and 9 months later). The data will be collected via online questionnaires. Patients’ treatment preferences, QoL, and self-reported health status will be compiled into an individual-level report containing graphics for quickly assessing key values. This report will be sent to the patient’s treating physician after the second measurement time point. Information about individual preferences and their fulfillment can help physicians to tailor treatment as closely as possible to patients’ preferences. Information about the patient’s health status and QoL can help physicians complement the impressions formed during consultations, providing them with a more comprehensive picture of the patients’ well-being. To examine the usefulness of this feedback for treating physicians (third objective, hypothesis 3), we will conduct a qualitative interview study with them. This protocol is based on the SPIRIT (Standard Protocol Items: Recommendations for Interventional Trials) reporting guidelines for study protocols [8] (Checklist 1). The quantitative longitudinal study will be embedded in the registry of the German Multi-Organ Auto-Immunity Network (GAIN)—a registry for individuals with congenital multi-organ autoimmune diseases, which has been documenting disease-specific patient data since 2020 [9]. The registry was designed to conduct embedded research studies whose output generates suggestions for improving patient care.

### Sample and Setting

Our target population consists of adult patients with multi-organ autoimmune diseases enrolled in the GAIN registry. GAIN patients are defined as having at least 2 different autoimmune or autoinflammatory diseases or carrying a possibly pathogenic mutation in a gene that can cause a multi-organ autoimmune disease, including asymptomatic carriers. The GAIN registry includes approximately 440 adult patients documented by 8 university hospitals in Germany, 1 in Italy, and 1 in Portugal (status as of May 2024). Most of the adult patients have been documented at the centers in Freiburg, Hannover, and Berlin (375/440, 85.2%). This study includes patients enrolled in the GAIN registry that are treated at these 3 centers.

### Inclusion and Exclusion Criteria

Inclusion criteria for participation in our study are that individuals are registered in the GAIN registry and have a multi-organ autoimmune disease as defined earlier. Furthermore, only individuals who have provided informed consent will be included in the study. Participants must be aged at least 18 years and have sufficient knowledge of the German language to answer the questionnaires. As the study asks about treatment preferences with respect to the documenting centers, participants must have been treated at the documenting centers at least once within the past 3 years.

### Sample Size

We aim to include as many patients for the longitudinal study as possible. Therefore, the 3 participating medical centers invite all eligible patients enrolled in the GAIN registry to participate. As part of their enrollment in the GAIN registry, the patients have provided consent to be invited to participate in future studies. Moreover, they regularly visit their medical centers (that invited them to participate in our study) for treatment. Given these circumstances, which are likely to increase motivation to participate in our study, and based on the high participation rate of a comparable patient population in a previous study [10], we expect a participation rate of 80% and a dropout rate of 15%. Consequently, we expect a sample size of 240 at the first measurement point (t1) and 200 at the last (t4). Regarding the aim of our study, the largest sample size is required for the psychometric analyses of our first and second goals. Because these exploratory analyses depend on many factors that are not known in advance, the recommendations in the literature should be considered guidelines only. Although large samples (ie, n>250) are recommended for these analyses [11], samples in the range of 100 to 200 should also be sufficient [12], as we expect a small number of resulting factors and moderate item commonalities (h²>0.50). Because the psychometric analyses will be conducted with the data collected at the first and second measurement time point, the expected sample size of ≥200 should provide a sufficiently large sample. This sample size should also be sufficiently large for testing hypothesis 1 of our third goal. A sample size of 200 allows for the detection of small-to-medium effects (Cohen *f*^2^=0.02 to 0.15) with a power of 0.80 and an α of .05. Specifically, if considering the multilevel structure is not necessary, effects of *f*^2^≥0.04 will be detectable with this sample size. However, if the examination of the intracluster correlation (ICC) indicates that the multilevel structure must be considered, then effects of *f*^2^≥0.07 (ICC=1% [13]) or *f*^2^≥0.12 (ICC=3% [14]) will be detectable.

### Recruitment

Before recruitment began, the medical centers at the universities of Freiburg, Hanover, and Berlin assessed which of their patients enrolled in the GAIN registry met the study’s inclusion criteria. Recruitment was then carried out by mail. To this end, the 3 centers sent letters from their leading physicians inviting their eligible patients to participate in the Qualy-GAIN study. These letters were accompanied by detailed study information; an informed consent form, which requested the patient’s email address because the longitudinal study is conducted using online questionnaires; and a prepaid envelope for the patients’ replies. For patients who have provided informed consent, participation in the study begins with receipt of an email containing a link to the first online questionnaire. In total, the questionnaire study includes 4 surveys conducted over a 9-month period. At baseline (t1), 3 months (t2), 6 months (t3), and 9 months (t4), participants will receive a link to an online questionnaire that will take approximately 30 minutes to complete.

### Measures

The focus of our study is on questionnaire development and exploratory research on patient preferences, QoL, and health status of patients with multi-organ autoimmune diseases. Relevant outcome measures for testing the hypotheses are disease-specific health status (self-reported), health-related QoL (generic and disease specific), as well as treatment preferences and their fulfillment.

Established questionnaires are used to measure subjective health status and health-related QoL with instruments from a pool of questionnaires used depending on the patient’s symptoms. The questionnaire package is adaptable and modular, so that, for example, a patient with skin and bowel symptoms will receive the specific questionnaires for assessing health-related QoL of patients with dermatological and bowel diseases. We use questionnaires that have already been used in other studies on our target group or similar rare disease patient groups: Short Form 12 Health Survey [15], EQ-5D [16], Functional Assessment of Chronic Illness Therapy**–**Fatigue [17], Chronic Obstructive Pulmonary Disease Assessment Test [18], Short Inflammatory Bowel Disease Questionnaire [19,20], Dermatology Life Quality Index [21,22], and Extra Short Musculoskeletal Function Assessment Questionnaire [23].

As described earlier, the instruments used to assess treatment preferences and their fulfillment will be newly developed. Close patient involvement in the preliminary studies (eg, individual interviews, Delphi study, and cognitive pretests) will ensure relevance and applicability for the target population. Table 1 shows the measurement of the outcome variables at the 4 measurement points of the longitudinal Qualy-GAIN study.

**Table 1.** Outcome variables of the longitudinal Qualy-GAIN study.

Outcome	Instrument	Baseline (t1)	3 months (t2)	6 months (t3)	9 months (t4)
Subjective health status					
Diagnosis and affected organs^a^	Self-developed	✓	✓	✓	✓
Strength of impairment caused by the affected organs	Self-developed	✓	✓	✓	✓
Overall health status	EQ-5D (visual analog scale; 1 Item) [16]	✓	✓	✓	✓
Impairment due to autoimmune disease and side effects of medication	Self-developed	✓	✓	✓	✓
Number of days of sick leave in the past year	Self-developed	✓	✓	✓	✓
Health-related QoL^b^ (generic)					
Physical and mental health–related QoL	Short Form 12 Health Survey [15]	✓	✓	✓	✓
Fatigue	Functional Assessment of Chronic Illness Therapy–Fatigue [17]	✓	✓	✓	✓
Health-related QoL (disease specific)^c^					
Affected organ: lung—QoL	COPD^d^ Assessment Test [18]	✓	✓	✓	✓
Affected organ: bowel—QoL	Short Inflammatory Bowel Disease Questionnaire [19,20]	✓	✓	✓	✓
Affected organ: skin—QoL	Dermatology Life Quality Index [21]	✓	✓	✓	✓
Affected organ: musculoskeletal system—QoL	German Short Musculoskeletal Function Assessment Questionnaire [22,23]	✓	✓	✓	✓
Treatment preferences and their fulfillment					
Treatment preferences	Self-developed	✓^e^	✓^f^	✓	✓
Perception of current treatment	Self-developed		✓^f^	✓	✓
Fulfillment of preferences^a^	Self-developed		✓^f^	✓	✓

a Fulfillment of preferences is inferred from the difference between the treatment preferences and current treatment.

b QoL: quality of life.

c Disease-specific quality of life measures were assessed only if the corresponding organ is affected.

d COPD: chronic obstructive pulmonary disease.

e All k=42 items were presented and psychometrically analyzed to form the instrument used at time point t2.

f Items were psychometrically analyzed to form the final instruments to measure treatment preferences and the perceived current treatment at the time points t3 and t4.

### Statistical and Qualitative Methods

Pseudonymized questionnaire data collected every 3 months will be used for data analysis. Psychometric analyses for our first goal include the determination of item characteristics, exploratory and confirmatory factor analyses, as well as internal consistency analyses. Our third goal comprises 3 hypotheses. To test hypothesis 1, linear mixed models will be used, in which self-reported health status and health-related QoL at the later time point (t3) are each predicted by the fulfillment of treatment preferences recorded at the earlier time point (t2). To minimize confounding, control variables (eg, age and sex) are included in the models. In addition, we will examine the ICC to decide whether to include the center of treatment as a random effect (random intercept) in the model. To test hypothesis 2, correlation analyses will be performed to assess the relationship of QoL and the self-reported health status with objective medical parameters from the GAIN registry. Hypothesis 3 will be evaluated on the basis of the qualitative interviews with treating physicians. As with the qualitative interviews with patients and physicians in the preliminary studies, the qualitative interviews used to evaluate hypothesis 3 will be analyzed using structuring qualitative content analysis according to the approach described by Kuckartz and Rädiker [24].

### Ethical Considerations

The study was approved by the Ethics Committee of the Albert-Ludwigs-University Freiburg (24-1064-S1; vote from May 23, 2024). Written informed consent is obtained from each participant (patient and physician) before participation. The privacy and confidentiality of participants’ data and identity are assured through processes described in a detailed data protection concept approved by the responsible data protection officer. Respondents will receive a shopping voucher worth €50 (US $59) as compensation for their participation.

## Results

The Qualy-GAIN study was funded from January 2023 to December 2025. Data collection for the prospective longitudinal study started in July 2024 and ended in June 2025. As of May 2025, data collection for 3 measurement points had been completed. Final data analyses began in summer 2025, with results expected to be submitted for publication in spring 2026.

## Discussion

### Anticipated Findings and Implications

The Qualy-GAIN study aims to contribute to improving the care and QoL of patients with multi-organ autoimmune diseases. This study uses a mixed methods approach that integrates quantitative and qualitative methods and is conducted by a multidisciplinary research team. Data from patients with multi-organ autoimmunity treated at 3 medical centers in Germany are collected in a longitudinal study with 4 measurement time points and will be linked to objective medical parameters from the GAIN registry.

Our first hypothesis (hypothesis 1) is that the fulfillment of preferences at an earlier stage is associated with a subsequently better self-reported health status and better health-related QoL. In line with previous studies [2,3] for other diseases, this is expected to improve our knowledge of the importance of patients’ preferences. We expect hypothesis 1 to be supported based on our data analysis. Our longitudinal dataset covering a relatively large sample of patients with multi-organ autoimmune diseases will enable us to detect small-to-medium effects of preference fulfillment on patient-reported outcomes.

We assume in the second hypothesis (hypothesis 2) that there are plausible correlations between self-assessed health status and QoL, and pathophysiology and physical symptoms. Given the complex and heterogeneous phenotypes of multi-organ autoimmunity [1], we anticipate only moderate correlations between subjective health status and objective clinical parameters.

Furthermore, we expect in hypothesis 3 that providing physicians feedback from patients regarding their health status, QoL, and preferences will be perceived as valuable for optimizing treatment and patient-centered care. Such feedback may strengthen patient-physician communication and enhance patient-centered care.

No serious risks for the patients are to be expected from participation in the study. It requires approximately half an hour to complete the online questionnaire at 4 measurement points within 9 months. If participants are unable to take part in some of the 4 online surveys for health reasons, they can skip their participation at these measurement points. Although this is associated with a loss of data for the study, information provided by the participants up to that point can still be used for the study. As no sensitive questions are asked in the online surveys, it can be assumed that the participating patients will not be exposed to any risk of harm by completing the online questionnaire.

As a result of the study, a questionnaire package will become available that can be used to assess health-related QoL, disease-specific health status, treatment preferences, and the fulfillment of treatment preferences. In addition, data collected with these instruments will provide the basis for testing the formulated hypotheses. Due to the voluntary nature of participation and the minimal burden on participation, we consider the risk of participants experiencing disadvantages as a result of participating in the project to be extremely low. Overall, we therefore believe that the benefits of the study are expected to outweigh the potential risks.

### Strengths and Limitations

A strength of the study is the prospective longitudinal design with multiple measurement points, which enables us to analyze the relationships between preference fulfillment, patient-reported outcomes, and clinical parameters. Another strength is that a relatively large sample of patients with an ultrarare disease who are treated in 3 medical centers can be examined in a longitudinal study.

Limitations of our study include the possibility of responder bias, as comparatively motivated and relatively healthy patients may respond disproportionately to the invitation to participate, leading to a selective sample of relatively healthy patients. Another limitation is that the patient-reported outcomes are collected 4 times at intervals of 3 months, whereas the measurement of objective medical parameters from the GAIN registry does not adhere to these time intervals. This may limit the comparability of the data and our ability to examine direct correlations between subjective and objective measures. To address these limitations, efforts were made to recruit representative patient populations through reminder strategies.

### Dissemination

We will disseminate our research results in various ways, including abstracts, posters, and presentations at conferences, as well as articles in peer-reviewed journals. The newly developed patient preference and fulfillment questionnaire will be published in open-access journals, making it available for future research and practice. Furthermore, we will make our results available to the physicians at the 3 centers involved and will invite participating patients to discuss the results with us.

### Conclusions

The procedure proposed here and the questionnaire set created may be transferred to other rare diseases to promote greater inclusion of patient-reported experience measures and PROMs in the everyday care of these patients. In case the questionnaires evaluated in this study prove to be reliable, valid, and practically useful, they can be transferred to the routine treatment of patients with multi-organ autoimmune diseases to improve their treatment and promote patient-centered care.

## Supplementary material

10.2196/77786 Checklist 1 SPIRIT (Standard Protocol Items: Recommendations for Interventional Trials) checklist.

## Abbreviations

GAIN: German Multi-Organ Auto-Immunity Network
ICC: intracluster correlation
PROM: patient-reported outcome measure
QoL: quality of life
SPIRIT: Standard Protocol Items: Recommendations for Interventional Trials
